# Accurate nanoelectrode recording of human pluripotent stem cell-derived cardiomyocytes for assaying drugs and modeling disease

**DOI:** 10.1038/micronano.2016.80

**Published:** 2017-03-13

**Authors:** Ziliang Carter Lin, Allister F. McGuire, Paul W. Burridge, Elena Matsa, Hsin-Ya Lou, Joseph C. Wu, Bianxiao Cui

**Affiliations:** 1 Department of Applied Physics, Stanford University, Stanford, CA 94305, USA; 2 Department of Chemistry, Stanford University, Stanford, CA 94305, USA; 3 Stanford Cardiovascular Institute, Stanford University, Stanford, CA 94305, USA; 4 Department of Pharmacology and Center for Pharmacogenomics, Northwestern University, Chicago, IL 60611, USA

**Keywords:** cardiomyocytes, drug screening, electrophysiology, human pluripotent stem cells, multielectrode array

## Abstract

The measurement of the electrophysiology of human pluripotent stem cell-derived cardiomyocytes is critical for their biomedical applications, from disease modeling to drug screening. Yet, a method that enables the high-throughput intracellular electrophysiology measurement of single cardiomyocytes in adherent culture is not available. To address this area, we have fabricated vertical nanopillar electrodes that can record intracellular action potentials from up to 60 single beating cardiomyocytes. Intracellular access is achieved by highly localized electroporation, which allows for low impedance electrical access to the intracellular voltage. Herein, we demonstrate that this method provides the accurate measurement of the shape and duration of intracellular action potentials, validated by patch clamp, and can facilitate cellular drug screening and disease modeling using human pluripotent stem cells. This study validates the use of nanopillar electrodes for myriad further applications of human pluripotent stem cell-derived cardiomyocytes such as cardiomyocyte maturation monitoring and electrophysiology-contractile force correlation.

## Introduction

From repairing damaged tissue *in vivo* to predicting drug efficacy, human pluripotent stem cells (hPSCs) have become a promising solution to many bottlenecks in human biological research^1–6^. There have been many efforts to generate cardiomyocytes (CMs) derived from human embryonic stem cells (hESCs) and human induced pluripotent stem cells (hiPSCs) due to the difficulty in obtaining human cardiac tissue by other methods^7^. In the last decade, efficient and reliable protocols, such as the embryoid body method and the monolayer differentiation method have been established to generate hPSC-CMs within 2 weeks and with >70% efficiency^8,9^. Critically, hPSC-CMs exhibit key cardiomyocyte properties, expressing the expected ion channels, exhibiting human cardiac-type action potentials, and containing functional sarcomeres.

Despite their potential, applications of hPSC-CMs have been hampered by the cells’ intrinsic heterogeneity and the quality of functional assays for monitoring their electrophysiology. First, hPSC-CMs are intrinsically heterogeneous, consisting of three subpopulations: atrial-, ventricular-, and nodal-like^10^. Furthermore, electrophysiology measurements have shown that the shapes of action potentials vary significantly between studies and within studies among different cell lines and differentiation methods^11,12^. Therefore, the high heterogeneity among hPSC-CMs requires that a large number of cells be analyzed to generate statistically meaningful conclusions.

The gold standard for measuring cardiomyocyte electrophysiology is manual patch clamp; however, this technique is laborious and time-consuming as the experimenter must address one cell at a time^13^. In addition, the whole culture, usually containing tens of thousands of cells, is discarded after patching only a few cells. This is a particular concern for hPSC-CMs because it takes considerable time and resources to generate the cells. Several automated patch clamp techniques have been developed to improve throughput, but these methods work only with suspended cells, and in general, cardiomyocyte ionic currents are not physiological when the cells are suspended. Multielectrode arrays and impedance measurements^14^ are high-throughput methods, but they do not capture the essential features of intracellular action potentials and lack one-to-one correspondence between a cell and the measurement. A high-throughput electrophysiology method suitable for hPSC-CM characterization has yet to be demonstrated.

The nanopillar electrode array platform recently developed by our group and others^15–17^ offers the opportunity to measure, in multiplex, intracellular action potentials of single cardiomyocytes in a beating, confluent monolayer. This measurement could yield the information relevant to pre-clinically monitor drug cardiotoxicity^3^, track cardiomyocyte maturation, and perform any variety of experiments enabled by multi-day current clamp-like measurements. In all these examples, it is vital to benchmark nanopillars against existing technology. The following work demonstrates the applicability of nanopillar electrodes to a variety of electrophysiological questions related to drug screening, disease modeling, and beyond. Here, we demonstrate the signal integrity of our platform compared to the patch clamp electrophysiology platform, its ability to resolve cardiomyocyte subtypes, and its utility in drug screening and disease modeling with hPSC-CMs.

## Materials and methods

### Nanopillar electrode device fabrication

The platinum nanoelectrode fabrication is similar to our previous work^15^. Briefly, Pt feedlines and pads were defined on quartz chips by a sequence of photolithography, metal deposition, and liftoff. Low-pressure chemical vapor deposition of 300 nm-thick SiO_2_ covered the metal lines and served as electrochemical insulation. Three-by-three arrays of nanoholes were then created on the SiO_2_ layer above each Pt pad by electron beam lithography (Raith, Dortmund, Germany) and reactive ion etch. The etching was selective for SiO_2_ so that it stopped upon reaching the Pt layer. Subsequently, Pt nanopillars were deposited onto these nanoholes by focused ion beam.

A small plastic tube was glued onto the center of each chip to form the culture chamber. Each device was then mounted and wire bonded onto a custom-designed printed circuit board to interface with the recording amplifiers. Before each cell culture, the devices were cleaned using oxygen plasma and then coated with Matrigel matrix (BD Bioscience, San Jose, CA, USA) diluted 20× in Dulbecco’s Modified Eagle’s Medium (Hyclone, GE Life Sciences, Logan, UT, USA) for 2 h, at 37 °C, before cell plating.

### Quartz nanopillar fabrication

Arrays of 100-nm-thick Cr nanodots were patterned on quartz coverslips by electron beam lithography, metal deposition, and liftoff. Using these nanodots as masks, quartz nanopillars were created by reactive ion etching with CHF_3_ and O_2_ chemistry. Each nanopillar array here is a 100×100 μm^2^ area spaced apart by 10 μm. The residual Cr was removed by CR-14 etchant (Transene, Danvers, MA, USA).

### Human embryonic stem cells and induced pluripotent stem cells

The details of the human pluripotent stem cell generation and cardiac differentiation were presented in our previous work^18^. The hESC line H7 was supplied by WiCell Research Institute^19^. For hiPSC generation, the protocols were approved by the Stanford University Human Subjects Research Institutional Review Board. With informed written consent, two 2-mm skin-punch biopsies were taken from each volunteer, diced with a scalpel, and digested with 1 mg mL^−1^ collagenase IV (Life Technologies, Carlsbad, CA, USA) for 2 h, at 37 °C. Fibroblasts were then grown in DMEM with GlutaMAX (Life Technologies) supplemented with 10% FBS (Life Technologies) on six-well plates coated with a 1:200 dilution of growth factor-reduced Matrigel (Corning, Tewksbury, MA, USA). All cell cultures were maintained in a 37 °C incubator with 5% CO_2_. Fibroblasts at passage 1 or 2 were seeded at 40 000 cells per well on Matrigel-coated six-well plates in E8 medium (E8 consisting of DMEM/F12 (Corning) with 20 μg mL^−1^ *Escherichia coli*-derived recombinant human insulin (Dance Pharmaceuticals/CS Bio, Brisbane, CA, USA), 64 μg mL^−1^ L-ascorbic acid 2-phosphate sesquimagnesium salt hydrate (Sigma-Aldrich, St Louis, MO, USA), 10.7 μg mL^−1^ Oryza sativa-derived recombinant human transferrin (Invitria/Sigma-Aldrich, Junction City, KY, USA), 14 ng mL^−1^ sodium selenite (Sigma-Aldrich), 100 ng mL^−1^ 154 amino acid *E. coli*-derived recombinant human FGF2 (Peprotech, Rocky Hill, NJ, USA), and 2 ng mL^−1^ 112 amino-acid HEK293-derived recombinant human TGFβ1 (Peprotech). At 24 h post-seeding, media was switched to E8 supplemented with four OSKM CytoTune-iPS 2.0 Sendai Reprogramming Kit viral particle factors (Life Technologies) at a multiplicity of infection of 7.5, as per the manufacturer’s instructions. For the first 7 days post-infection, cultures were maintained in E8 supplemented with 100 nM hydrocortisone (Sigma-Aldrich) and 200 μM sodium butyrate (Sigma-Aldrich), provided fresh every 24 h. At day 7, cells were passaged using TrypLE (Life Technologies) and seeded into Matrigel-coated 6-well plates with E7N media (E8 deprived of TGFβ1; and supplemented with 200 μM sodium butyrate). Two micromolar thiazovivin (Selleck Chemicals, Houston, TX, USA) was added for the first 24 h after passage. Media was provided fresh every 24 h and switched to E8 on day 20 post-infection. On days 20–25 post-infection, single colonies with pluripotent stem cell-like morphology were picked and seeded at a density of 1 colony per well, in E8 supplemented with 2 μM thiazovivin for 24 h after picking. Confluent wells were expanded into six-well plates by passaging 1:1, 1:4, 1:6, 1:8, and 1:12 using a 7 min incubation with 0.5 mM EDTA (Life Technologies) in D-PBS without Ca^2+^/Mg^2+^ (Life Technologies) and at room temperature.

### Cardiac differentiation and culture

hESCs and hiPSCs were differentiated in either RPMI+B27-ins medium or CDM3 medium. RPMI+B27-ins medium consists of RPMI 1640 medium supplemented with 2% B27 without insulin (Life Technologies). CDM3 consists of RPMI 1640 medium (Life Technologies), 500 μgmL^−1^ *O. sativa*-derived recombinant human albumin (Sigma-Aldrich), and 213 μg L^−1^ L-ascorbic acid 2-phosphate (Sigma-Aldrich). From day 0 to day 2, the medium was supplemented with 6 μM CHIR99021 (LC Laboratories, Woburn, MA, USA). On day 2, the medium was changed to one that was supplemented with 2 μM Wnt-C59 (Selleck Chemicals). From day 4 on, the medium was changed to one without any supplements. On days 15–20, the cells were plated on the nanopillar devices with the same medium. During the first 24 h after plating on the devices, the human embryonic stem cell-derived cardiomyocytes (hESC-CMs) were incubated with 20% fetal bovine serum, while the human induced pluripotent stem cell-derived cardiomyocytes (hiPSC-CMs) were incubated with 10 μM ROCK inhibitor (Y27632). The medium was changed daily.

### Electrophysiology recording

All recordings were performed at 37 °C outside the incubator by a 60-channel voltage amplifier (MEA1060-Inv-BC, Multi-Channel Systems, Reutlingen, Germany) at a 5 kHz sampling rate. All cells were recorded in their respective culture media. The electroporation pulse consisted of 100 consecutive biphasic and symmetric square pulses, each with a 400 μs period and 3.5 V amplitude. Recordings were resumed less than 10 s after electroporation to avoid amplifier saturation. Nanopillar electrode devices were reused over multiple cultures using a cleaning protocol of trypsinization, DI water, and oxygen plasma. Simultaneous whole-cell current and voltage clamps were performed at room temperature (Axon Multiclamp 700B, Molecular Devices, Sunnyvale, CA, USA). Patch pipettes had a resistance of 2–4 MΩ. The intracellular solution contained the following (in mM): 140 KCl, 10 NaCl, 10 HEPEs, and 1 EGTA (pH 7.3); the extracellular bath solution was the culture medium. The data were sampled at 10 kHz and low-pass filtered at 3 kHz (Axon Digidata 1440A, Molecular Devices).

### Electron microscopy

hESC-CMs cultured on the quartz nanopillars were fixed with 2% glutaraldehyde and 4% paraformaldehyde in 0.1 M sodium cacodylate buffer (pH 7.4) and post-fixed in 1% osmium tetroxide in the same buffer at 4 °C for 1 h. Chemicals were from Electron Microscopy Sciences. After rinsing, the sample was dehydrated in a graded ethanol series and dried in a liquid CO_2_ critical point dryer (Autosamdri 815b, Tousimis, Rockville, MD, USA). The sample was then sputter-coated with 4 nm Au/Pd and imaged by electron microscopy (Strata 235DB, FEI, Hillsboro, OR, USA).

### Drug assays

Each drug was first dissolved in dimethyl sulfoxide (DMSO) to 10 mM and subsequently diluted in the culture medium and administered to the cell cultures to reach the targeted concentration. The final concentration of DMSO in the cell culture ranged between 0.01 and 0.05%. For each drug experiment, an intracellular recording was first performed in the absence of any drugs for control purposes. The culture was then returned to the incubator for 2 h to allow for recovery. The cell culture was then incubated with the drug for 10 min, and a second intracellular recording was performed. The culture medium was then completely replaced, and the cells’ attachment and proliferation were monitored daily.

## Results

### Intracellular recording of human cardiomyocytes and nanopillar interface

**Figure 1:**
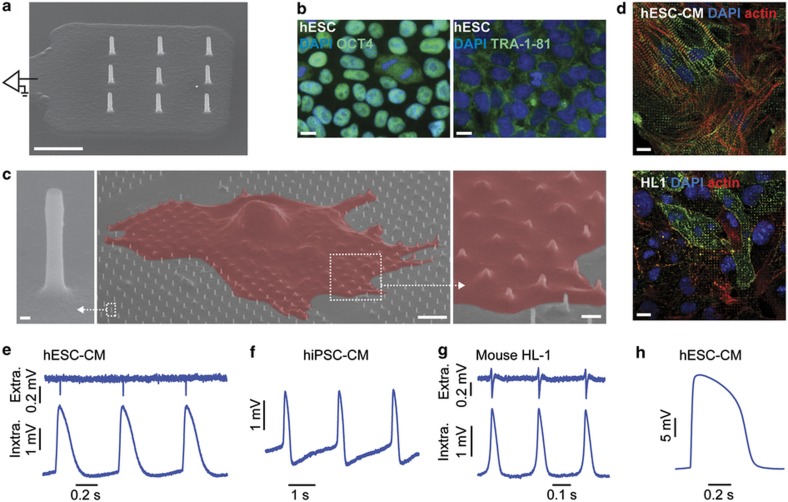
Recording of human embryonic stem cell-derived cardiomyocyte action potentials. (**a**) Scanning electron micrograph of a nanopillar electrode array on top of a multielectrode array architecture. Scale bar: 2 μm. (**b**) Immunofluorescent staining of OCT4, and TRA-1–81 in the hESCs confirms their pluripotency. Scale bars: 10 μm. Scanning electron micrograph of a human embryonic stem cell-derived cardiomyocyte (hESC-CM) interfacing with a large array of quartz nanopillars of similar geometry as in **a**. Magnified view shows the cell-nanopillar interface. Scale bars: left, 100 nm; middle, 5 μm; right, 1 μm. (**d**) Immunofluorescent staining of actin and α-actinin in the hESC-CMs and the mouse HL-1 cells shows co-localization with nanopillar arrays. Scale bar: 10 μm. (**e**–**g**) Intracellular recording of action potentials of hESC-CM (**e**), human induced pluripotent stem cell-derived cardiomyocyte (hiPSC-CM) (**f**), and mouse HL-1 cells (**g**). (**h**) The largest action potential signal recorded by nanopillars of amplitude 25.15 mV.

The recording electrodes consist of 3×3 arrays of vertical Pt nanopillars deposited on top of a Pt pad (Figure 1a). Each nanopillar is ~200 nm in diameter and 1.5 μm in height, and the spacing between adjacent nanopillars is 2 μm. The footprint of each array (4×4 μm^2^) is smaller than that of a single cell. The substrate surface is insulated by a layer of chemical-vapour-deposited SiO_2_ so that only the vertical nanoelectrodes are capable of recording the signal. Each pad is connected to an external stimulator and an amplifier for electroporation and recording, respectively.

The cardiomyocytes used in this study were derived from human pluripotent stem cells. We used both human embryonic stem cell-derived cardiomyocytes (hESC-CMs) and human induced pluripotent stem cell-derived cardiomyocytes (hiPSC-CMs). Before differentiating the hESCs into cardiomyocytes, we verified their pluripotency by the immunofluorescent staining of OCT4 (also known as POU5F1) and TRA-1–81 (Figure 1b), which are pluripotency markers. After differentiating these hESCs into cardiomyocytes, we observe α-actinin and actin in striated patterns (Figure 1c). The pluripotency of the hiPSCs was also confirmed (Supplementary Figure S1).

We then connected our electrode device to an amplifier and recorded the cardiac action potentials in the form of extracellular spikes (Supplementary Figure S2). To achieve intracellular recording, we sent in a train of voltage pulses via the nanopillars to create nanometer sized holes in the basal cell membrane surrounding the nanopillars^15,16^. After electroporation, we observed a dramatic amplitude increase and a signal shape that matches that of patch clamp-recorded intracellular action potentials. The magnitude of this increase in amplitude is variable and depends upon how the cells engulf the nanopillars^16^. The method of intracellular recording after electroporation is applicable for hESC-CMs (Figure 1e), hiPSC-CMs (Figure 1f), and mouse HL-1 cells (Figure 1g). For the hESC-CMs, we achieved a maximum intracellular action potential amplitude of 25.15 mV (Figure 1h), which is only approximately three times attenuated from the full amplitude recorded by patch clamp^18^ and larger than any previous chip-based nanoelectrode recording of mouse HL-1 cardiomyocytes^15,16^. With a peak-to-peak noise level of 30 μV_pp_, we achieved a signal-to-noise ratio of 838 over a 5 kHz bandwidth. Our previous result was a signal-to-noise ratio of 590 with mouse HL-1 cardiomyocytes and the same type of electrodes^15^.

To study the cardiomyocyte-nanopillar interface, we fabricated large arrays of vertical quartz nanopillars of similar geometry and spacing and cultured hESC-CMs (age ~20 days after differentiation) on top of them (Figure 1c). Because the quartz arrays were functionalized with Matrigel in the same manner applied to the recording devices and both are high Young’s modulus materials, we reasoned that the cardiomyocytes behaved similarly across the substrates. Using electron microscopy, we observed that the cardiomyocytes engulfed the nanopillars underneath them (Figure 1c). At the same time, we did not observe any mechanical penetration of the cells by these nanopillars, which agrees with our previous findings and those of others^15,16,20^.

### Nanopillar recordings are accurate compared with patch clamp

**Figure 2:**
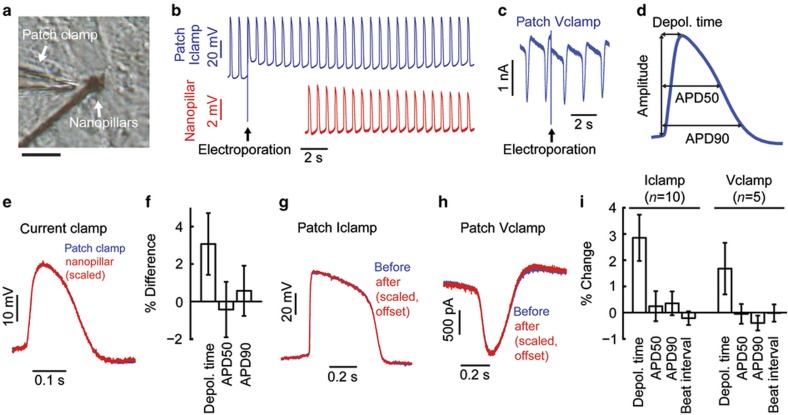
Nanopillars provide accurate recording compared with patch clamp. (**a**) Bright field inverted microscope image of a human embryonic stem cell-derived cardiomyocyte (hESC-CM) simultaneously recorded by the nanopillar electrode and a patch clamp electrode (Supplementary Movie). Scale bar: 20 μm. (**b**) Simultaneous recording of an hESC-CM with patch current clamp. (**c**) Recording of an hESC-CM in patch voltage clamp before and after nanopillar electroporation. (**d**) Definition of action potential amplitude, depolarization time, APD50, and APD90. (**e**) Overlay of hESC-CM action potentials recorded by a nanopillar electrode and a patch clamp electrode. (**f**) Statistics of percentage difference between nanopillar electrode and patch clamp electrode recordings (*n*=6). (**g** and **h**) Overlay of hESC-CM action potentials recorded by a patch clamp electrode in current clamp mode (**g**) and voltage clamp mode (**h**) before and after nanopillar electroporation. (**i**) Percentage change in electrophysiological parameters recorded by patch whole-cell current clamp (*n*=10) and voltage clamp (*n*=5) after nanopillar electroporation. All error bars denote standard error of the mean (s.e.m.).

Next, to verify that our nanopillars record accurate signals, we compared nanopillar and whole-cell patch clamp recordings. To do so, we simultaneously recorded from a single cardiomyocyte by the nanopillar electrodes and a patch clamp electrode (Figure 2a and Supplementary Video 1). Immediately after the nanopillar electroporation, the patch current clamp revealed a depolarization of the resting membrane potential due to the pores created on the cell membrane (Figure 2b). Since this electroporation was transient and the pores sealed over time, the resting membrane potential gradually returned to the pre-electroporation level. At the same time, these pores allowed the nanopillars to gain electrical access to the cell interior while they remained physically outside of the cell^15,16^. The pore sealing manifested in the decay of the recorded signal amplitude, similar to our previous observations with Pt nanopillar^15^ and iridium oxide nanotube electrode recordings of mouse HL-1 cardiomyocytes^16^. The same result was observed in the patch voltage clamp mode (Figure 2c). The nanopillar electroporation created pores that led to an increase in the patch clamp-monitored leakage current. We then analyzed key electrophysiological parameters such as depolarization time and action potential duration at 50% repolarization (APD50) and at 90% repolarization (APD90; Figure 2d). An overlay of the signal recorded by the nanopillar and the patch clamp shows that the action potential shapes match identically after scaling the amplitude of the nanopillar-recorded action potentials (Figure 2e). Statistics show that there is less than a 3% difference in the depolarization time and an insignificant difference in the APD50 and APD90 in the nanopillar recording (Figure 2f). We note that the cells’ beating was coordinated between the flat area of the device and the nanopillar-covered areas and that action potentials recorded on the nanopillars were indistinguishable from routine patch clamp recordings from the same cell type (data not shown). Thus, we posit that the cells’ electrophysiology is not significantly perturbed.

Furthermore, we investigated whether this nanopillar electroporation is invasive to the recorded cardiomyocytes. Previously, we demonstrated that nanopillar recording is minimally invasive on a time scale of days and allows the multi-day recording of the same cell^15,16^. However, questions remain as to whether this electroporation method is invasive on a shorter time scale and whether it alters the electrophysiology of the cells under test. To answer this question, we compared the action potentials recorded by the patch clamp electrode before and after nanopillar electroporation. In both the current clamp and voltage clamp modes, the action potential shapes recorded before and after electroporation are identical after scaling (Figures 2g and h). The post-electroporation amplitudes must be scaled to match the pre-electroporation amplitudes due to the poration-induced shunt resistance. Statistics shows that in this case, too, there is less than a 3% prolongation in the depolarization time, and there are no changes in the APD50 and APD90 (Figure 2i). We believe this slower depolarization time results from the resting potential depolarization caused by electroporation. Since the sodium channel current is dependent upon the membrane potential, the elevation of the membrane potential leads to a smaller sodium channel current and slower depolarization time.

### Cardiomyocyte subpopulation and maturation

**Figure 3:**
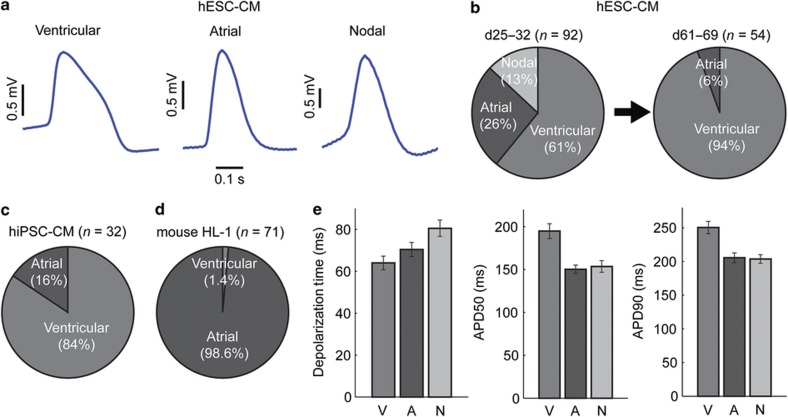
Electrophysiological parameters for human embryonic stem cell-derived cardiomyocyte (hESC-CM) subtypes. (**a**) Example intracellular recording of action potentials from the hESC-CM subtypes. (**b**) At day 25–32, the hESC-CM culture contains a mixed population of ventricular, atrial, and nodal-like cells. The percentage of ventricular-like cells increases as the cultures age to day 61–69. (**c**) Human induced pluripotent stem cells (hiPSC)-derived cardiomyocytes also consist of more ventricular cells than atrial cells. (**d**) The HL-1 mouse cardiomyocyte cell line consists predominately of atrial cells. (**e**) Statistics of depolarization time, APD50, and APD90. Error bars denote standard error of the mean (s.e.m.).

Using our method, a single cell contributes to a given signal in the majority of cases, and we can distinguish all three cardiomyocyte subtypes within a population based on the recorded action potential shapes (Figure 3a)^21^. We identified the ventricular-like action potential by its rapid depolarization to a peak, followed by a slow repolarization phase and subsequent fast repolarization. The atrial-like cells were classified based on a single-phase repolarization. We identified the nodal-like cardiomyocytes based on the two depolarization phases in their action potentials, namely, the existence of a slow depolarization period before a rapid one. We observed that most of the cells within the population at day 25–32 were ventricular-like cells (61%), with atrial-like cells composing most of the remaining population (26%) followed by nodal-like cells (13%; Figure 3b). As the culture aged to days 61–69, we found that the population shifted toward mostly ventricular-like cells (94%). Our observations agree with the literature results recorded with patch clamp^22^. We also classified the hESC-CM population in a chemically defined medium CDM3 (Supplementary Figure S3) compared to the RPMI+B27 medium. We again observed that most of the cells in the population were ventricular-like. We have also recorded hiPSC-CMs from healthy individuals and found that >80% of the recorded cells were ventricular-like (Figure 3c). As a comparison, we observed that the mouse HL-1 population consisted of atrial cardiomyocytes to an overwhelming extent independent of the culture age (Figure 3d), which agrees with the fact that HL-1 is a cell line derived from a mouse atrial cardiomyocyte tumor lineage^23^.

To further validate the integrity of our platform for biological measurements, we took hESC-CMs that were 25–32 days old and analyzed the depolarization time, APD50, and APD90 (Figure 3e). We found that ventricular-like cells have a shorter depolarization time than atrial-like ones, which, in turn, are faster than nodal-like ones. On the other hand, the ventricular-like cells have much longer APD50 and APD90. These results agree well with our previous study of the same cell type in CDM3 medium^18^ and the literature using patch clamp electrophysiology^24^.

### Drug screening assay and disease modeling

**Figure 4:**
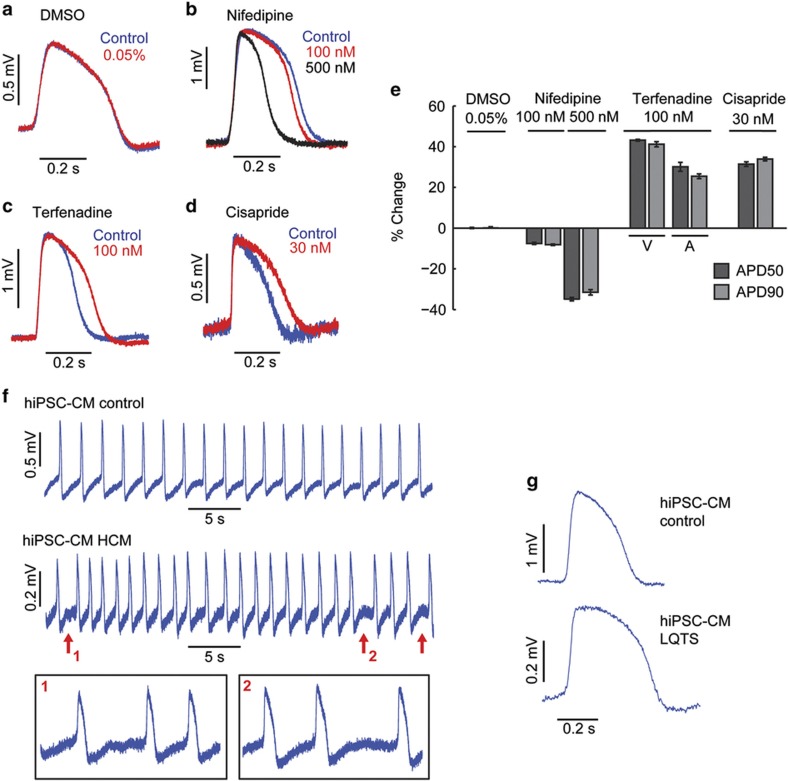
Drug screen assays and diseased cardiac cell modeling. (**a**) The DMSO carrier does not alter the human embryonic stem cell-derived cardiomyocyte (hESC-CM) action potential shape. (**b**) Nanopillars show hESC-CM action potential duration reduction by nifedipine at 100 and 500 nM. (**c** and **d**) Nanopillars show hESC-CM action potential duration prolongation by terfenadine (**b**) and cisapride (**c**). All recordings are scaled to match the amplitude of the respective control recordings and overlaid on their rising edges in **a**–**d**. (**e**) Statistics of percentage change for APD50 and APD90 for different drugs. All assays were performed on the ventricular-like hESC-CM cells, except for terfenadine, where the atrial-like cells were also assayed. (**f**) Nanopillar recording of human induced pluripotent stem cell-derived cardiomyocytes (hiPSC-CMs). Top panel shows a recording from a healthy individual, and the bottom panel shows a recording from a patient with HCM. (**g**) Top nanopillar recording is representative of a healthy individual, while the bottom recording shows the characteristic long action potential duration of long QT syndrome (LQTS) cardiomyocytes.

After verifying that our nanopillar recordings are accurate and minimally invasive, we applied them in multiple drug tests to demonstrate their potential in pharmacological research. We tested three different drugs with the hESC-CMs. In each of these experiments, the intracellular action potentials were first recorded in the absence of drugs as a control. The cardiomyocytes were then returned to the incubator to allow the resealing of transient membrane pores. Subsequently, a specific drug concentration was added into the culture medium, and a second intracellular recording was performed to examine the drug effect on action potentials. Because all of the drugs tested were dissolved in DMSO and subsequently diluted in the culture medium, we first tested how this DMSO carrier affects the cell action potentials. We found that the DMSO in the culture medium with a final concentration up to 0.05% w/w results in no change in the recorded action potentials (Figures 4a and e). The first drug we tested, nifedipine, blocks L-type calcium channels, and our recordings showed that this effect reduces the action potential duration in a dose-dependent manner (Figures 4b and e). On the other hand, terfenadine, a potassium channel blocker, lengthened the action potential duration (Figures 4c and e) and had a different effect on ventricular-like and atrial-like cells. The third drug, cisapride, a serotonin receptor agonist, also lengthens the action potential duration but was potent at a lower concentration than terfenadine (Figures 4d and e). These results are consistent with patch clamp measurements with the same compounds^25–27^.

To demonstrate the capability of our technique in a clinical electrophysiology assay, we applied our nanopillars to the recording of human cardiomyocytes with two different cardiac disorders. These cardiomyocytes were derived from induced pluripotent stem cells that were reprogrammed from human patient fibroblasts. The first disease we studied was hypertrophic cardiomyopathy (HCM). The healthy cardiomyocytes’ action potentials show a regular beating interval, whereas we observed two types of abnormalities in the diseased cells (Figure 4f). The action potentials exhibit arrhythmia and also delayed afterdepolarization, as indicated by the red arrows. The second disease we studied was long QT syndrome (LQTS), which is characterized by the delayed repolarization and therefore prolongation of the action potential and may be studied using hiPSC-CMs^28^. Comparison of the recordings from the control cells and LQTS-afflicted cells reliably demonstrates the electrical manifestation of the disease when recorded by nanopillars (Figure 4g).

## Discussion

To fulfill their potential in various areas, human stem cell-derived cardiomyocytes must be paired with appropriate measurement tools. In the particular cases of screening for cardiotoxicity and monitoring maturation, these tools must be able to measure the action potential of cardiomyocytes with high throughput and a high signal-to-noise ratio in their preferred culture environment. For most studies, this entails an adherent cell culture where the cells can form a tissue-like monolayer and spontaneously beat both physically and electrically. We have demonstrated herein that the nanopillar electrode array platform is compatible with cardiomyocyte culture and retains high signal integrity compared with the gold standard of patch clamp electrophysiology. As the nanopillar electrode arrays record intracellular action potentials, they enable distinction between the various cardiomyocyte subtypes that are inevitably present in stem cell-derived populations. This constitutes a significant advance beyond planar electrode arrays, which require hPSC-CM populations of carefully validated purity^29^, while retaining the throughput of multielectrode arrays. Finally, the nanopillar electrodes do not require signal averaging and can thus measure arrhythmic behavior in diseases such as LQTS and hypertrophic cardiomyopathy, lending the platform to patient-in-a-dish type applications^30^.

The ongoing development of hPSC-CMs and electrophysiological tools to assess them could lead to a new paradigm for preclinical drug safety and efficacy screening in cardiology as well as an improved understanding of their development and disease.
