# Supplementary material for: Accurate nanoelectrode recording of human pluripotent stem cell-derived cardiomyocytes for assaying drugs and modeling disease
Source: Microsyst Nanoeng. 2017 Mar 13;3:16080. doi: 10.1038/micronano.2016.80 (PMC6444980; doi:10.1038/micronano.2016.80)
Supplement: Supplementary Figures [file micronano201680-s1.pdf]

Supplementary file

Accurate nanoelectrode recording of human pluripotent stem cell-derived cardiomyocytes for assaying drugs and modeling disease

Ziliang Carter Lin[1], Allister F McGuire[2], Paul W Burridge[3,4], Elena Matsa[3], Hsin-Ya Lou[2], Joseph C Wu[3] and Bianxiao Cui[2]

Microsystems & Nanoengineering (2017) **3,** 16080; doi:10.1038/micronano.2016.80; Published online: 13 March 2017

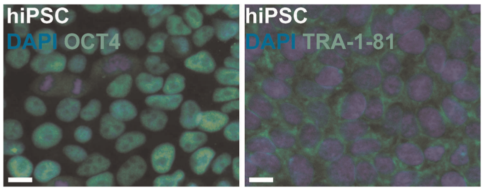

Figure S1 Immunofluorescent staining of hiPSCs. Staining of OCT-4 and TRA-1-81 in the hiPSCs confirms their pluripotency. Scale bars: 10 μm.

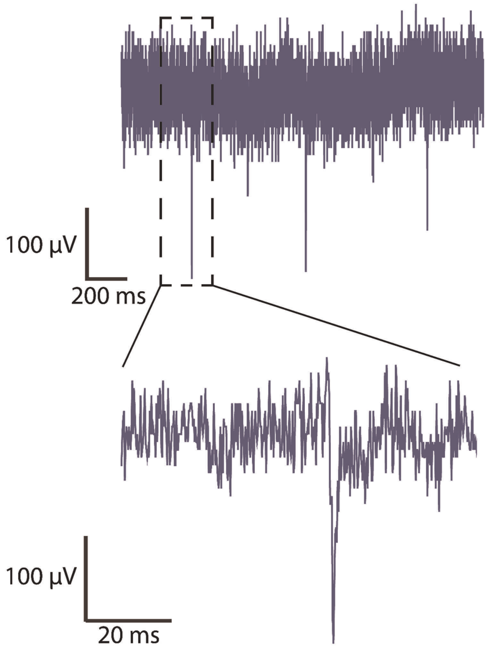

Figure S2 Extracellular hESC-CM action potentials. hESC-CM action potentials recorded by Pt nanopillars before electroporation.

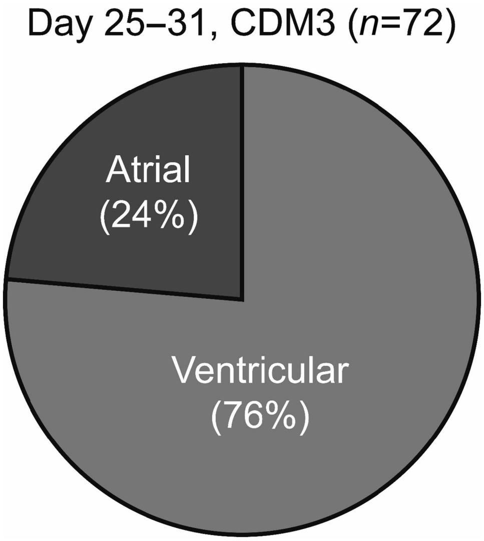

Figure S3 hESC-CM population subtypes in CDM3. hESC-CMs cultured in CDM3 for 25–31 days after differentiation show a mixed population of atrial-like and ventricular-like cells as determined by intracellular action potential shapes on Pt nanopillars.

[1]Department of Applied Physics, Stanford University, Stanford, CA 94305, USA; [2]Department of Chemistry, Stanford University, Stanford, CA 94305, USA; [3]Stanford Cardiovascular Institute, Stanford University, Stanford, CA 94305, USA and [4]Department of Pharmacology and Center for Pharmacogenomics, Northwestern University, Chicago, IL 60611, USA.
Correspondence: Bianxiao Cui (bcui@stanford.edu)
